# A Decision-Based Modified Total Variation Diffusion Method for Impulse Noise Removal

**DOI:** 10.1155/2017/2024396

**Published:** 2017-04-27

**Authors:** Hongyao Deng, Qingxin Zhu, Xiuli Song, Jinsong Tao

**Affiliations:** ^1^School of Information & Software Engineering, University of Electronic Science and Technology of China, Chengdu 611731, China; ^2^College of Computer Engineering, Yangtze Normal University, Chongqing 408000, China; ^3^School of Computer Science and Technology, Chongqing University of Posts and Telecommunications, Chongqing 400065, China; ^4^School of Electrical Engineering, Wuhan University, Wuhan 430072, China

## Abstract

Impulsive noise removal usually employs median filtering, switching median filtering, the total variation *L*_1_ method, and variants. These approaches however often introduce excessive smoothing and can result in extensive visual feature blurring and thus are suitable only for images with low density noise. A new method to remove noise is proposed in this paper to overcome this limitation, which divides pixels into different categories based on different noise characteristics. If an image is corrupted by salt-and-pepper noise, the pixels are divided into corrupted and noise-free; if the image is corrupted by random valued impulses, the pixels are divided into corrupted, noise-free, and possibly corrupted. Pixels falling into different categories are processed differently. If a pixel is corrupted, modified total variation diffusion is applied; if the pixel is possibly corrupted, weighted total variation diffusion is applied; otherwise, the pixel is left unchanged. Experimental results show that the proposed method is robust to different noise strengths and suitable for different images, with strong noise removal capability as shown by PSNR/SSIM results as well as the visual quality of restored images.

## 1. Introduction

Images often contain impulsive noise, which may arise from transmission errors, malfunctioning camera photo sensors, optic imperfections, or poor illumination. The sources of image disturbances include lightning, strong electromagnetic interferences caused by faulty high voltage powerline insulation, car starters, and unprotected electric switches. These noise sources generate short high-energy pulses. As a result, corrupted images contain sparsely occurring isolated pixels, for example, white and black points, and grayscale points that differ significantly from their neighbors.

Reducing such noise is critical in many applications, such as pattern recognition, image identification, and image fusion. Noise-free medical images can significantly assist in the diagnosis of a disease; for example, imaging systems can detect metabolic changes in the hippocampus of Alzheimer's disease patients, when assessing the risk of dementia. Various methods have been proposed over the past several decades to produce clean images from noisy versions.

The simple and direct methods are the median (MED) filter [1] and its variants. A MED filter replaces every observed pixel with the median pixel within its neighborhood; this neighborhood is also called sliding window. MED and MED variants output a median pixel but there are differences between them. The Adaptive Median Filter (AMF) [2] adopts a dynamic sliding window whose size depends adaptively on local noise density. The Weighted Median Filter (WMF) [3] employs a weighted adjustment window that differentially duplicates the pixels within a current window. The Center-Weighted Median Filter (CWMF) [4], in contrast, considers the number of copies and sets the largest number for the center pixel. The Adaptive Center-Weighted Median Filter (ACWMF) [5] combines the AMF and CWMF techniques. These median filtering (MF) methods introduce too much smoothing and blur visual features extensively. Moreover, they are only suited to low density noise; these methods do not make a distinction between noisy pixels and noise-free pixels.

Apart from MF, there is another family of methods termed switching filtering (SF). Different from MF, SF makes a distinction between noisy pixels and noise-free pixels, containing a detection and a removal module. The former identifies the noisy pixels; the latter applies an algorithm to corrupted pixels to obtain an estimate of the noise-free pixels. In SF, impulse detection is the core technique. If the detection module fails to identify corrupted pixels, they will be left unchanged, resulting in poor restoration image. If the detection module correctly classifies a noisy pixel but also identifies noise-free pixels as noisy, much of the image details will be lost. Therefore, accurate and complete identification determines the quality of filtered images.

Currently, many methods are used to identify impulses. Three ways are summarized. The first compares a noisy image with an estimate of its noise-free image pixel by pixel. If the absolute difference of paired pixels is larger than a predefined threshold, the corresponding pixel is declared as corrupted; otherwise, it is declared as noise-free. To obtain an estimate of the noise-free image, a median and weighted median-based impulse detector is devised in [6]; the ACWMF is used in [7]; an improved ACWMF is proposed in [8] where if the noise density is larger than 30%, the sliding window shape of the IACWMF is changed; otherwise, it reverts to ACWMF; a nonlocal median filter is utilized in [9]; a spatial inverse filter is employed in [10]. Estimation of a noise-free pixel determines whether the pixel is corrupted, not the final output. The second method to identify impulses exploits the local neighborhood to design detectors statistically, for example, the Rank-Ordered Absolute Differences (ROAD) [11], the Directional Absolute Relative Differences (DARD) [12], and using the first eight minimum aggregated intensities of the pixels within a sliding window that contains twenty-five pixels [13]. The third method uses artificial intelligence techniques, such as an Artificial Neural Network (ANN) [14], Neurofuzzy Network (NFN) [15], or Support Vector Machine (SVM) [16].

Apart from MF and SF, variational and partial differential equations use a variational energy minimization model to obtain a solution. One of these is the Rudin-Osher-Fatemi (ROF) model proposed by Rudin et al. for edge-preserving image restoration [17]. However, ROF method exhibits staircase effects in restored images [18]. To avoid such artifacts, various modified methods have been developed such as adaptive or weighted total variation regularization [19, 20]. Despite their usefulness, the methods usually do not work with high density noise because they treat corrupted and uncorrupted pixels uniformly.

Inspired by switching filtering and variational methods, in this paper, a new method is proposed to reduce impulsive noise consisting of two schemes. One is for the salt-and-pepper nose model, and the other is for the random valued impulse model. In the first scheme, pixels are divided into corrupted and uncorrupted. The modified total variation diffusion is only applied on the corrupted pixels while the uncorrupted pixels are left unchanged. In the second scheme, the pixels are divided into corrupted, uncorrupted, and possibly corrupted. For corrupted pixels, modified total variation diffusion is used to cope with them; for possibly corrupted pixels, weighted total variation diffusion is used; and uncorrupted pixels are left unchanged. Experimental results show that the proposed method is extremely effective not only for salt-and-pepper noise but also for random valued impulses and is well suited for the medical and biomedical imaging fields such as [21, 22].

*Our Contributions*. In this work, we propose a new method to reduce impulsive noise for grayscale images. The five main contributions are as follows:

- A fast and efficient statistical detector is proposed to identify salt-and-pepper noise. When the intensity of a current pixel is 0 or 255, the detector works out the number of the pixels within the current neighborhood, such that each pixel must satisfy the condition that the absolute difference between it and the current pixel is larger than a predefined threshold. If the number is larger than 3, the current pixel is declared as corrupted; otherwise, it is a noise-free pixel. This detection method is very effective despite its simplicity.
- Pixels are divided into categories based on different noise characteristics. If an image is corrupted by salt-and-pepper noise, the pixels are divided into corrupted and noise-free; if the image is corrupted by randomly valued impulses, the pixels are divided into corrupted, noise-free, and possibly corrupted.
- Pixels falling into different categories are processed differently. If a pixel is declared as corrupted, modified total variation diffusion is applied; if the pixel is declared as possibly corrupted, weighted total variation diffusion is applied; otherwise, the pixel is left unchanged.
- An accelerated scheme is proposed for the salt-and-pepper noise model that replaces the initialization noisy image with an evolved intermediate image obtained by a new mean filter. The accelerated scheme significantly decreases the number of iterations.
- The proposed method is robust to different noise strengths and images, with a strong noise removal capability. Experiments show that, for salt-and-pepper noise model, denoising results obtained by the proposed method significantly outperform those obtained by the MED filter [1] and the common Switching Median Filter (SMF) [6]; the proposed method also outperforms the results obtained by the Spectral Gradient Method (SGM) [7]. In the random valued impulse model, denoising results obtained by the proposed method significantly outperform the traditional total variation diffusion (TVD) and the New Selective Degenerate Diffusion (NSDD) [23] method.

*Organization*. The rest of the paper is organized as follows. In Section 2, eight existing diffusion methods are introduced.  Section 3 describes the details of noise removal. Experimental results and comparisons are formulated in Section 4. And conclusions are drawn in Section 5.

## 2. Existing Diffusion Methods

Diffusion is an approach to image denoising. Let *u* be an image intensity function,* c* be a spatially varying diffusion coefficient, *t* be the time step, and div and ∇ denote divergence and gradient operators, respectively. The universal diffusion framework is

(1) $$\begin{array}{c} u_{t}=div\left(\;c\cdot \nabla u\;\right). \end{array}$$

Based on different designs for the diffusion coefficient, many diffusion equations have been proposed. Eight classical diffusivity coefficients are detailed as follows.

*Linear Diffusion [24]*. Emerging from the heat conduction in physical sciences, linear diffusion is also called heat diffusion. The diffusivity function of it is a constant, which is usually set to 1; that is,

(2) $$\begin{array}{c} c\left(\;x,y,t\;\right)=1. \end{array}$$

The corresponding diffusion equation (or heat equation) is

(3) ∂∂tux,y,tΔux,y,t=∂2ux,y,t∂x2+∂2ux,y,t∂y2,

where the symbol Δ is the Laplacian operator. As in [24], linear diffusion is equivalent to a smoothing process with a Gaussian kernel. Therefore, linear diffusion has a drawback similar to Gaussian filtering, which is the uniform filtering of local signal features and noise, and leads to smooth image details.

*Perona and Malik Diffusion [25]*. PM diffusion is a nonlinear conduction process, where the diffusion can take place with variable conduction in order to control the smoothing effect. There are two kinds of diffusivity functions in PM diffusion, given by

(4) c1x,y,t=exp⁡−∇ux,y,tλ2,

(5) c2x,y,t=11+∇ux,y,t/λ2,

where the diffusion constant *λ* is a gradient modulus threshold that controls the conduction. The diffusion coefficient in PM process is a decreasing function of the gradient of the signal. As can be seen from (4) or (5), the diffusivity function conducts along edges; therefore, the PM diffusion method preserves edges and controls smoothing. Some drawbacks and limitations however are that the diffusivity functions are very sensitive to noise, and further the PM diffusion equation is ill-posed [26].

*Total Variation Diffusion*. TV diffusion is also a nonlinear conduction process, proposed as a smoothing measurement for images in regularized models. The diffusion coefficient is unbounded as

(6) $$\begin{array}{c} c\left(\;x,y,t\;\right)=\frac{1}{\left|\nabla u\left(x,y,t\right)\right|}. \end{array}$$

In the TV process, the diffusivity function prevents edges from diffusing. Therefore, TV diffusion plays an edge-preserving role. Experiments show that it effectively recovers piecewise constant signals [27].

*Charbonnier Diffusion*. Charbonnier diffusion coefficient employs a half-quadratic function [28]:

(7) $$\begin{array}{c} c\left(\;x,y,t\;\right)=\frac{1}{\sqrt{1+{\left(\left|\nabla u\left(x,y,t\right)\right|/\lambda \right)}^{2}}}. \end{array}$$

The diffusion function is well posed because it is strictly convex. Similar to the PM and TV processes, it also preserves edges. 

*Weickert Diffusion*. Weickert diffusion is anisotropic diffusion. It employs a structure tensor to identify features such as corners or measure the local coherence of structures [29]. The specific diffusivity function is

(8) cx,y,t=1,∇ux,y,t=01−exp⁡−3.3148∇ux,y,t/λ2,∇ux,y,t>0.

In contrast to PM diffusion, Weickert diffusion preserves the boundaries between different regions; however, it usually creates ring artifacts on smooth regions. 

*FAB Diffusion [30]*. Forward-and-backward diffusion is also nonlinear diffusion. The diffusivity function is

(9) $$\begin{array}{c} c\left(\;x,y,t\;\right)=\frac{1}{\sqrt{1+{\left(\left|\nabla u\right|/k_{f}\right)}^{2}}}-\frac{\alpha }{1+{\left(\left|\nabla u\right|/k_{b}\right)}^{2}}. \end{array}$$

This coefficient is locally adjusted according to image features such as edges and textures and can switch the diffusion process from a forward to a backward mode according to a given set of criteria. In this equation, the parameter *k*_*f*_ limits the gradients to be smoothed, while the parameter *k*_*b*_ defines the range of backward diffusion. 

*NSDD Diffusion [23]*. NSDD diffusion method is a modification on the Selective Degenerate Diffusion (SDD), and the acronym NSDD stands for New Selective Degenerate Diffusion. The diffusion flow of it is

(10) $$\begin{array}{c} \frac{\partial u}{\partial t}=g_{n}\left(\;{ENI}_{P}\left(\;u,w,T\;\right)\;\right)\left|\;\nabla u\;\right|div\left(\frac{\nabla u}{\left|\nabla u\right|}\right), \end{array}$$

where *g*_*n*_(ENI_*P*_(*u*, *w*, *T*)) is the scaling function and ENI_*P*_(*u*, *w*, *T*) is associated with a noise detector; the scaling function of SDD is *g*_*n*_(∇*u*).

## 3. The Proposed Noise Removal Method

In this section, the details of the proposed method are introduced.  Section 3.1 introduces the salt-and-pepper noise model and the random valued impulse model and their denoising structures.  Section 3.2 formulates the noise detection methods.  Section 3.3 describes the processing techniques of noise suppression. The acceleration of the proposed method in the salt-and-pepper noise model is discussed in Section 3.4, and the computational complexity in the random valued impulse model is analyzed in Section 3.5.

### 3.1. Noise Model and Denoising Structure

As mentioned in the Introduction, in this paper, we only focus on impulse noise removal, assuming 8-bit grayscale image representation. Let *u*_*i*,*j*_ represent the pixel at the position (*i*, *j*) in an image; a contamination image can be modeled as

(11) $$\begin{array}{c} u_{i,j}=\left\{\begin{array}{cc} v_{i,j} & \text{with probability }\pi \\ o_{i,j} & \text{with probability }1-\pi , \end{array}\right. \end{array}$$

where *o*_*i*,*j*_ and *v*_*i*,*j*_ denote the original noise-free pixel and the contaminative pixel, respectively. In this model, the intensity of *v*_*i*,*j*_ is a random variable. If *v*_*i*,*j*_ takes on the value 0 or 255 with equal probability, then it is a salt-and-pepper noise model, denoted by SPM; if *v*_*i*,*j*_ takes on any value from the range [0, 255], then it is random valued impulse model, denoted by RDM. The corresponding denoising methods associated with the two noise models are introduced in the next subsection.

**Figure 1:**
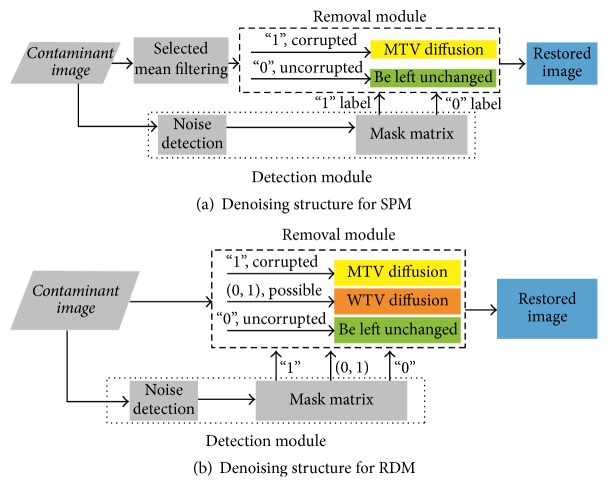
The structure of impulse noise removal.

The denoising structures are shown in Figure 1. As seen in inset (a) in Figure 1, the denoising structure in SPM consists of a noise detection module, a noise removal module, and an acceleration module. In the noise detection stage, a detector is used to identify noisy pixels, dividing pixels into noisy and noise-free pixels. Based on these detection results, a mask matrix is built where each entry is a binary label indicating whether or not the pixel is corrupted. In the noise removal stage, modified TV (MTV) diffusion is applied on the noisy pixels, while the noise-free pixels are left unchanged. To achieve better results, diffusion operations are iteratively implemented following the mask matrix instructions. To reduce the number of iterations, the initialization image in the iterative process is an evolved intermediate image, rather than a noisy image, created by a selected mean filter, that is, the acceleration module.

Inset (b) in Figure 1 shows the denoising structure in RDM. In contrast to SPM, the pixels are divided into three categories, that is, noise-free, noisy, and possibly noisy pixels. Pixels falling into different categories are processed differently. If a pixel is declared as noisy, it is processed by MTV diffusion; if the pixel is declared as a possibly noisy one, it is processed by weighted TV (WTV) diffusion; otherwise, it is noise-free and is left unchanged.

### 3.2. Noise Detection Methods

There are two noise detection methods corresponding to SPM and RDM, respectively. Both are based on the local neighborhood. Let *u*_*x*,*y*_ be referred to as the current pixel centered in the sliding window; then, the neighborhood of the current pixel is defined by

(12) Nx,yR=i,j:x−i≤R,y−j≤Rfor  x,y∈Ω,

where *R* is a positive integer representing the neighborhood radius and *Ω* is the image domain. From the definition, a neighborhood contains (2*R* + 1) × (2*R* + 1) pixels.

*The Noise Detection Method in SPM*. In this method, pixels with intensity 0 or 255 are the noisy pixel candidates. For each candidate, denoted by *u*_*x*,*y*_, the absolute differences of intensities between it and every pixel within the neighborhood are calculated, and then the number of pixels, whose absolute difference is larger than a predefined threshold value, is determined, expressed as

(13) $$\begin{array}{c} m_{x,y}=\#\left\{\;u_{i,j}:\left(\;i,j\;\right)\in N_{x,y}\left(\;R\;\right),\left|\;u_{x,y}-u_{i,j}\;\right|>T\;\right\}, \end{array}$$

where *m*_*x*,*y*_ denotes the number of the pixels that satisfy the threshold condition, the symbol # denotes the cardinality of the set, and *T* is the predefined threshold value.

The number *m*_*x*,*y*_ is used to determine whether a current candidate is corrupted as a noise-free image usually consists of local smoothly varying areas separated by edges, while salt-and-pepper noise takes a value substantially larger or smaller than its neighbors. The concrete reasons are twofold. (i) If *u*_*x*,*y*_ is a noisy pixel surrounded by a flat region, *m*_*x*,*y*_ is very large with high probability, whereas if *u*_*x*,*y*_ is a noise-free pixel, *m*_*x*,*y*_ is very small with high probability. (ii) If *u*_*x*,*y*_ is a noisy pixel riding on edges, *m*_*x*,*y*_ is large with high probability, whereas if *u*_*x*,*y*_ is a noise-free pixel, *m*_*x*,*y*_ is small with high probability. Therefore, *m*_*x*,*y*_ can be used to determine whether *u*_*x*,*y*_ is a noisy pixel or not by a given threshold, denoted by thr. The determining function is

(14) $$\begin{array}{c} f\left(\;x,y\;\right)=\left\{\begin{array}{cc} 1, & \text{if }m_{x,y}>thr\\ 0, & \text{otherwise}. \end{array}\right. \end{array}$$

In this equation, if *f*(*x*, *y*) = 1, then candidate *u*_*x*,*y*_ is corrupted; otherwise, it is uncorrupted. When all candidates are complete, a binary mask matrix can be built; each entry is either 1 or 0.

*The Noise Detection Method for RDM*. In this method, all the pixels are the noisy pixel candidates. We employ the Rank-Ordered Absolute Differences (ROAD) method [11] to assign a fuzzy index to each pixel. Based on these indices, the corresponding pixel is judged as corrupted or uncorrupted. Let *u*_*x*,*y*_ be the current pixel; the ROAD of *u*_*x*,*y*_ is defined by

(15) $$\begin{array}{c} {ROAD}_{\alpha }\left(\;x,y\;\right)=\overset{\alpha }{\underset{k=1}{\sum }}r_{k}\left(\;x,y\;\right), \end{array}$$

where 2 ≤ *α* ≤ (2*R* + 1)×(2*R* + 1) and

(16) rkx,y=kth  smallest  ux,y−ui,j,for  i,j∈Nx,yR.

Then, the index of *u*_*x*,*y*_ is

(17) hx,y=0,ROADαx,y≤T1ROADαx,y−T1T2−T1,T1<ROADαx,y<T2,1,ROADαx,y≥T2,

where *T*_1_ and *T*_2_ are predefined threshold parameters. *h*(*x*, *y*), for  (*x*, *y*) ∈ *Ω*, is a determining function, which divides pixels into three categories. If *h*(*x*, *y*) = 0, *u*_*x*,*y*_ is declared as noise-free; if *h*(*x*, *y*) = 1, *u*_*x*,*y*_ is declared as noisy; otherwise, it is a possibly noisy pixel.

### 3.3. Modified Diffusion Methods

Pixels falling into different categories are processed in different ways. In SPM, only noisy pixels are processed. Let *D* ⊂ *Ω* denote the noisy pixel domain; modified TV diffusion is applied on the noisy pixels, given by

(18) $$\begin{array}{c} MTV\left(\;u\;\right)=\iint_{D}\left|\;\nabla u\;\right|dx\;dy. \end{array}$$

Applying Euler-Lagrange method to (18), the modified TV descent flow can be written as

(19) ∂∂tux,y,t=div1∇uβ·∇u,for  x,y∈Dux,y,0=u0,

where *u*_0_ denotes the noisy image, ${\left|\nabla u\right|}_{\beta }=\sqrt{{\left|\nabla u\right|}^{2}+\beta }$, and *β* is a positive lifting parameter in order to avoid |∇*u*| vanishing.

Three categories of pixels, noise-free, noisy, and possibly noisy, are considered in RDM. For noise-free and noisy pixels, processing is the same as in SPM. Weighted TV diffusion, however, is applied on possibly noisy pixels. The weight assigned to the current pixel *u*_*x*,*y*_ is obtained by

(20) $$\begin{array}{c} h_{x,y}^{w}\left(\;n\;\right)=h\left(\;x,y\;\right)\exp\left(\;-n+1\;\right), \end{array}$$

where *n* = 1,2,… denotes the *n*th iteration, *h*_*x*,*y*_^*w*^(*n*) denotes the weight at the *n*th iteration, and

(21) $$\begin{array}{c} 0<h\left(\;x,y\;\right)=\frac{{ROAD}_{\alpha }\left(x,y\right)-T_{1}}{T_{2}-T_{1}}<1. \end{array}$$

The diffusion operation is implemented iteratively. A scaled version of the current residual error, *h*_*x*,*y*_^*w*^(*n*)(*u*_0_ − *u*), is also considered. Synthesizing the different processing approaches, the descent flow associated with RDM is given by

(22) ∂∂tux,y,t=div1∇uβ·∇u,for  x,y∈Θvhx,ywdiv1∇uβ·∇u+hx,ywu0−u,for  x,y∈Θp,ux,y,0=u0,

where Θ^*v*^ and Θ^*p*^ denote the noisy pixel domain and the possibly noisy pixel domain, respectively.

Discretization of the divergence div(∇*u*/|∇*u*|_*β*_) must be discussed, and discrete schemes for (19) and (22) are detailed. By using the central finite difference technique and half-pixel resolution, the divergence at the current pixel *u*_*i*,*j*_ is expressed as

(23) div∇u∇uβ=∂∂xux∇uβ+∂∂yuy∇uβ≃ux∇uβi+1/2,j−ux∇uβi−1/2,j+uy∇uβi,j+1/2−uy∇uβi,j−1/2,

where *u*_*x*_ and *u*_*y*_ are the first-order derivatives in the *x* and *y* directions, respectively.

**Figure 2:**
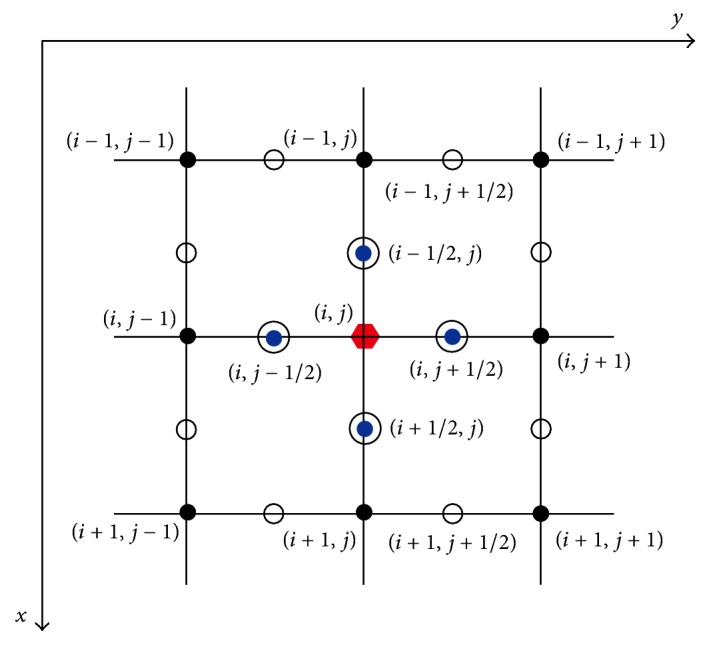
The representation of discretization at a half-pixel resolution.

Figure 2 illustrates the discretization at a half-pixel resolution. At the half-pixel (*i*, *j* + 1/2), the three formulas hold as follows:

(24) ∇ui,j+1/2uxi,j+1/2,uyi,j+1/2uyi,j+1/2ui,j+1−ui,juxi,j+1/2ui+1,j+1/2−ui−1,j+1/22=ui+1,j+1+ui+1,j−ui−1,j−ui−1,j+14.

Thus,

(25) uy∇uβi,j+1/2uyi,j+1/2uxi,j+1/22+uyi,j+1/22+β=C1,i,jui,j+1−ui,j,

where

(26) $$\begin{array}{c} C_{1,\left(i,j\right)}=\frac{1}{\sqrt{{\left(u_{i+1,j+1}+u_{i+1,j}-u_{i-1,j}-u_{i-1,j+1}\right)}^{2}/16+{\left(u_{i,j+1}-u_{i,j}\right)}^{2}+\beta }}. \end{array}$$

Similarly, at the other three half pixels,

(27) uy∇uβi,j−1/2=C2,i,jui,j−ui−1,j,ux∇uβi+1/2,j=C3,i,jui+1,j−ui,j,ux∇uβi−1/2,j=C4,i,jui,j−ui−1,j,

where

(28) C2,i,j=1ui+1,j−1+ui+1,j−ui−1,j−1−ui−1,j2/16+ui,j−1−ui,j2+β,C3,i,j=1ui+1,j+1+ui,j+1−ui+1,j−1−ui,j−12/16+ui+1,j−ui,j2+β,C4,i,j=1ui,j+1+ui−1,j+1−ui,j−1−ui−1,j−12/16+ui−1,j−ui,j2+β.

Substituting (25) and (27) to (23), the divergence div(∇*u*/|∇*u*|_*β*_) at the current position (*i*, *j*) can be discretized as

(29) $$\begin{array}{c} {div\left(\frac{\nabla u}{{\left|\nabla u\right|}_{\beta }}\right)}_{i,j}=\overset{4}{\underset{k=1}{\sum }}\left[\;C_{k,\left(i,j\right)}\left(\;P_{k,\left(i,j\right)}-u_{i,j}\;\right)\;\right], \end{array}$$

where *P*_*k*,(*i*,*j*)_, for  *k* = 1,2, 3,4, denote the 4 neighbors of the current pixel; that is,

(30) P1,i,j=ui,j+1;P2,i,j=ui,j−1;P3,i,j=ui+1,j;P4,i,j=ui−1,j.

Therefore, for flow (19) in SPM, the explicit discrete scheme is

(31) ui,jn+1=ui,jn+Δt∑k=14Ck,i,jnPk,i,jn−ui,jn,if  i,j∈D;

for flow (22) in RDM, the explicit discrete scheme is

(32) $$\begin{array}{c} u_{i,j}^{n+1}=\left\{\begin{array}{cc} u_{i,j}^{n}+\left(\Delta t\right)\overset{4}{\underset{k=1}{\sum }}\left[C_{k,\left(i,j\right)}^{n}\left(P_{k,\left(i,j\right)}^{n}-u_{i,j}^{n}\right)\right], & \text{if }\left(i,j\right)\in \Theta^{v}\\ u_{i,j}^{n}+\left(\Delta t\right)h_{i,j}^{w}\left(n\right)\overset{4}{\underset{k=1}{\sum }}\left[C_{k,\left(i,j\right)}^{n}\left(P_{k,\left(i,j\right)}^{n}-u_{i,j}^{n}\right)\right]+\left(\Delta t\right)h_{i,j}^{w}\left(n\right)\left[{\left(u_{0}\right)}_{i,j}^{n}-u_{i,j}^{n}\right], & \text{if }\left(i,j\right)\in \Theta^{p}. \end{array}\right. \end{array}$$

In (31) and (32), Δ*t* denotes the time marching step size and the superscript *n* = 1,2,…, *N* denotes the *n*th iteration where *N* denotes the desirable number of iterations.

### 3.4. Acceleration in SPM

In (19), the initialization image is a noisy image; that is, *u*(*x*, *y*, 0) = *u*_0_. To decrease the number of iterations, a noisy image *u*_0_ is replaced by an evolved intermediate image. In this paper, a selected mean switching filter was used to obtain the evolved intermediate image.

The selected mean switching filter consists of noise detection and noise removal. The noise detection employs the SPM detection method, and noise removal takes the arithmetical mean of uncorrupted pixels within a neighborhood as the current output. The details of the latter process are discussed as follows.

Let *D*^c^ ⊂ *Ω* be the noise-free set where the superscript *c* is the complementary operator; the noise-free neighborhood is defined as

(33) Nx,y0R0=i,j:i,j∈Nx,yR0  &  i,j∈Dc,

where *N*_*x*,*y*_(*R*^0^) is the neighborhood of the current pixel *u*_*x*,*y*_ and *R*^0^ is the filtering window radius.

Using the arithmetical mean operator, denoted by the symbol* mean*, the selected mean filtering is

(34) u^x,y=meanui,j:i,j∈Nx,y0R0,for  x,y∈D.

When complete, an evolved intermediate image is obtained, denoted by ${\hat{u}}_{0}$. Therefore, the accelerated diffusion method can be written as

(35) ∂∂tux,y,t=div1∇uβ·∇u,for  x,y∈Dux,y,0=u^0.

**Figure 3:**
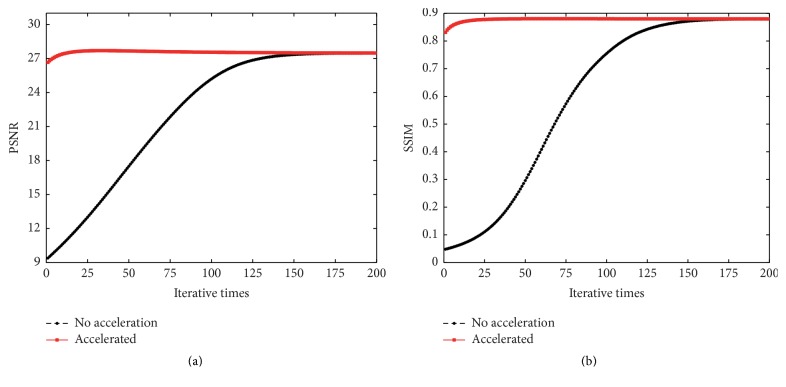
Comparing the iterative progress before and after acceleration when *π* = 50% and Δ*t* = 0.8. The test image is Straw, sized 1024 × 1024 pixels.

Next, the acceleration performance is validated. For ease of formulation, (19) is termed nonaccelerated and (35) is called accelerated. The two methods are applied to the same test image Barbara, sized 512 × 512, with different density noise. For fixed density noise, when PSNR (similar to SSIM) reaches the peak value in the iterative process, the corresponding iterative times are noted. The numbers of iterations associated with different density noise are shown in Table 1. For example, when noise density *π* = 50%, at least 180 iterations were needed to achieve a peak value of PSNR for the nonaccelerated image, while for the accelerated image, 43 were required. Almost the same results were obtained for the SSIM metric.  Figure 3 illustrates the acceleration process comparing iterative progress when noise density *π* = 50%. From Table 1 and Figure 3, we can draw a conclusion that the number of iterations for an accelerated image was about a fourth of that for the nonaccelerated one, under the same conditions, Δ*t* = 0.8.

### 3.5. Computational Complexity in RDM

The computational complexity of the proposed method in RDM is discussed in this subsection. The time expended during the judgment operation was not considered, so these values were excluded from calculations. Let there be *M* pixels in an image. As calculating each ROAD (15) requires *O*((2*R* + 1)^2^log_2_(2*R* + 1)^2^) operations, using quick sort, the number of operations required to build the mask matrix can be bounded by *O*(*M* · (2*R* + 1)^2^log_2_(2*R* + 1)^2^). In each iteration of the removal stage, obtaining an estimate of the noise-free pixel requires *O*(41) operations if a pixel is declared as corrupted and requires *O*(47) operations if the pixel is declared as possibly corrupted, as in (32). Let there be *m*^*v*^ corrupted pixels and *m*^*p*^ possibly corrupted pixels; obtaining an estimate of the noise-free image requires *O*(41*m*^*v*^ + 47*m*^*p*^) operations per iteration. Therefore, when *K* iterations are completed, the number of operations for the total complexity is

(36) OM·2R+12log22R+12+OK41mv+47mp.

**Figure 4:**
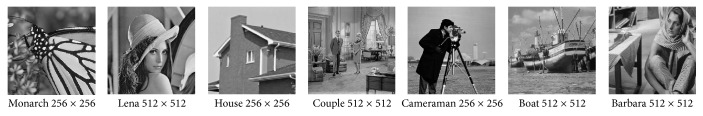
The miscellaneous noise-free images associated with the group Γ_1_.

To better illustrate the computational complexity in RDM, the image Monarch (shown in Figure 4) was taken to test, sized 256 × 256, with 0.05 density salt-and-pepper noise. Using Matlab implementation, on eight machines with Intel(R) Core(TM) i7-4702MQ CPU at 2.20 GHz, took 62.4213 seconds when *K* = 120 and *R* = 2.

## 4. Experiments

In our experiments, two metrics were used to evaluate noise removal performance, the Peak Signal-to-Noise Ratio (PSNR) and the Structural Similarity Index Measure (SSIM), introduced briefly in this section.

### 4.1. Two Metrics

The PSNR measurement is based on pixel intensity errors between the noise-free and the restored images. The calculation form of PSNR is given by

(37) $$\begin{array}{c} PSNR=10\;{log}_{10}\left(\;\frac{255^{2}}{{\left|u\right|}^{-1}{\left\|u-\hat{u}\right\|}_{F}^{2}}\;\right), \end{array}$$

where |·| is the cardinality of image, ‖·‖_F_ denotes the Frobenius norm, and *u* and $\hat{u}$ are the noise-free and the restored images, respectively.

The SSIM measurement is based on structural similarity. Its computation involves two blocks, denoted by *y*_1_ and *y*_2_. Let *μ*_*y*_1__ and *μ*_*y*_2__ be the mean values of *y*_1_ and *y*_2_, respectively, *σ*_*y*_1__ and *σ*_*y*_2__ be the variances, and *σ*_*y*_1_*y*_2__ be the covariance; thus, the calculation of SSIM is as follows:

(38) $$\begin{array}{c} SSIM\left(\;y_{1},y_{2}\;\right)=\frac{\left(2\mu_{y_{1}}\mu_{y_{2}}+c_{1}\right)\left(2\sigma_{y_{1}y_{2}}+c_{2}\right)}{\left(\mu_{y_{1}}^{2}+\mu_{y_{2}}^{2}+c_{1}\right)\left(\sigma_{y_{1}}^{2}+\sigma_{y_{2}}^{2}+c_{2}\right)}, \end{array}$$

where *c*_1_ and *c*_2_ denote two stabilization variables. Actually, the measurement is the mean SSIM that yields mean value of the structural similarity between the blocks of noise-free image *u* and restored image $\hat{u}$. In this paper, the SSIM is referred to as the mean SSIM.

### 4.2. Setting Parameters

*The Parameters in SPM*. A total of six parameters are set in the proposed SPM method. As seen in inset (a) in Figure 1, three parameters (*R*, *T*, thr) are used in the noise detection module; two parameters (Δ*t*, *N*) are used in the noise removal module; and the parameter *R*^0^ is used in the acceleration module. For all noise levels, (*R*, *T*, thr) were set to 2, 60, and 3, respectively. The time step size Δ*t* was set to 0.8, which conforms with the CFL criteria. The desirable number of iterations *N* depends on the noise density *π* and the time step size Δ*t*. The higher the noise density or the larger the time step size is, the more the iteration is needed. The values of *N* in our experiments are shown in Table 1 when Δ*t* is set to 0.8. The parameter *R*^0^ depends on noise densities. The value of this parameter should be greater than or equal to the number of noisy pixels within the sliding window [31]. The setting values of *R*^0^ in our experiment can be seen in Table 2.

*The Parameters in RDM*. There are six parameters to be set in the proposed RDM method. As seen in inset (b) in Figure 1, four parameters (*R*, *α*, *T*_1_, *T*_2_) are used in the noise detection module, and two parameters (Δ*t*, *N*) are used in the noise removal module. In our experiments, the density noise is not larger than 60%, and the four parameters (*R*, *α*, *T*_1_, *T*_2_) were set to 2, 14, 150, and 320, respectively, and the time step size Δ*t* was set to 0.5. Similar to the SPM method, the desirable number of iterations *N* depends on the noise density *π* and the time step size Δ*t*. In RDM, the values of *N* for RDM can be seen in Table 3 when Δ*t* = 0.5.

All the parameters used in our experiments are summarized in Table 4.

### 4.3. Results and Comparisons

*Experimental Results and Comparisons in SPM*. A test set was built to evaluate the proposed method in SPM, which was a combination of three groups, denoted by Γ = {Γ_1_, Γ_2_, Γ_3_}. Each group Γ_*k*_ contained corrupted versions of seven images, with different density salt-and-pepper noise. The original noise-free images associated with Γ_1_, Γ_2_, and Γ_3_ are shown in Figures 4, 6, and 8, respectively. They include miscellaneous, texture, and biomedical images taken from the CVG-Granada database.

**Figure 5:**
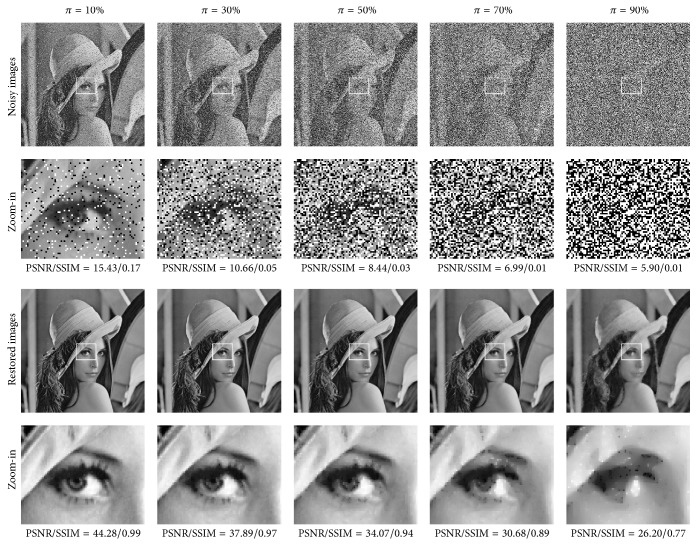
Visual results for the Lena image on five different noise levels in SPM.

**Figure 6:**
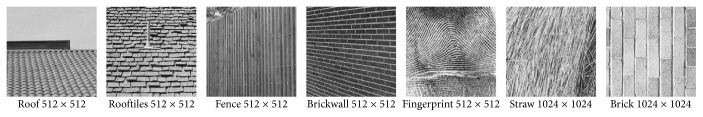
The texture noise-free images associated with the group Γ_2_.

**Figure 7:**
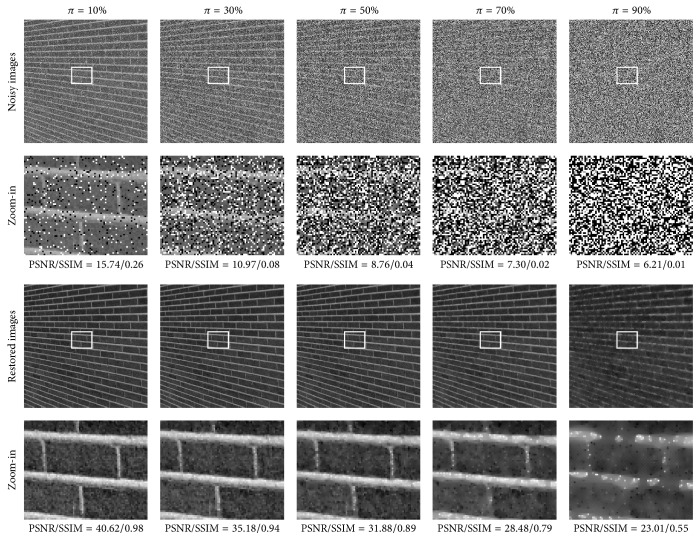
Visual results for the Brickwall image on five different noise levels in SPM.

**Figure 8:**
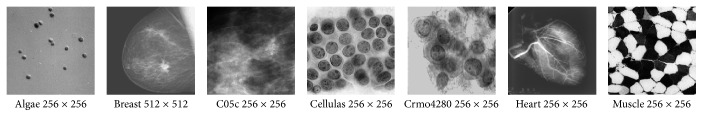
The noise-free biomedical images associated with the group Γ_3_.

**Figure 9:**
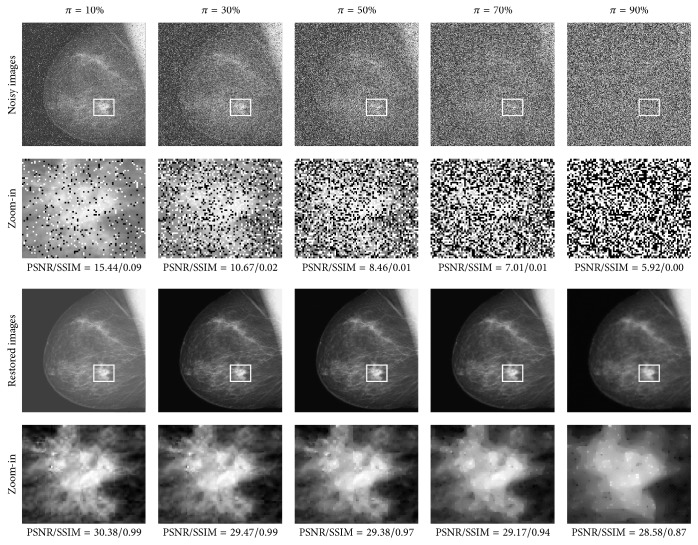
Visual results for the Breast image on five different noise levels in SPM.

The proposed SPM method was applied on the test set Γ. The parameters were set in accordance with the SPM field shown in Table 4. The PSNR and SSIM results associated with Γ_1_, Γ_2_, and Γ_3_ are reported in Tables 5, 6, and 7, respectively. Moreover, the visual results and zoom-in for the three images (i.e., Lena, Brickwall, and Breast) are shown in Figures 5, 7, and 9, respectively.

**Figure 10:**
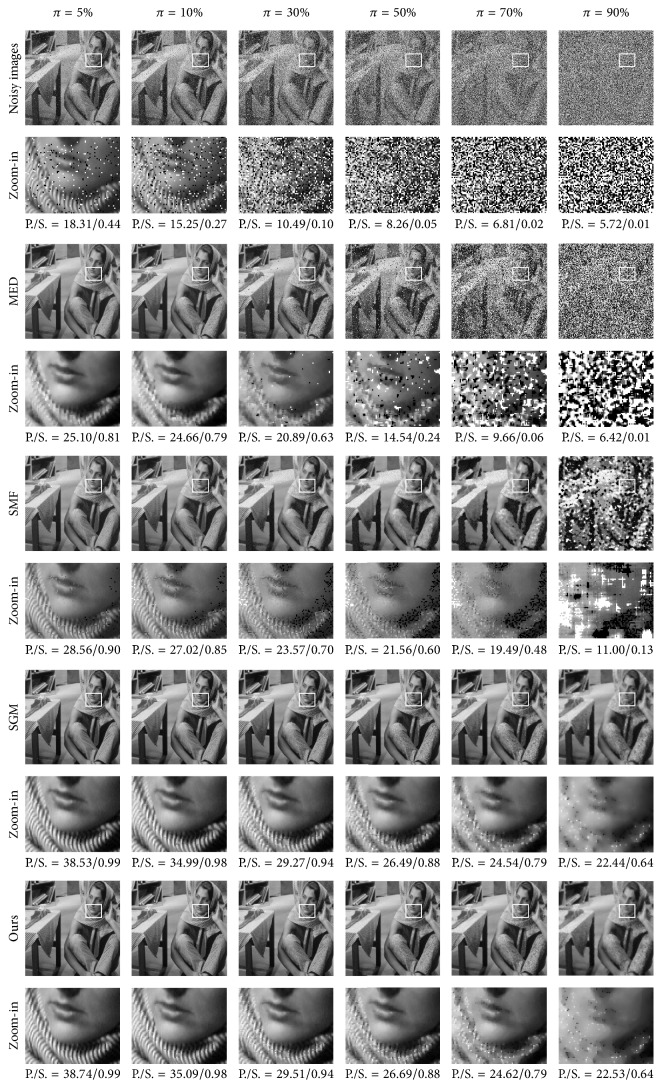
Visual results from different methods on the Barbara image in SPM.

**Figure 11:**
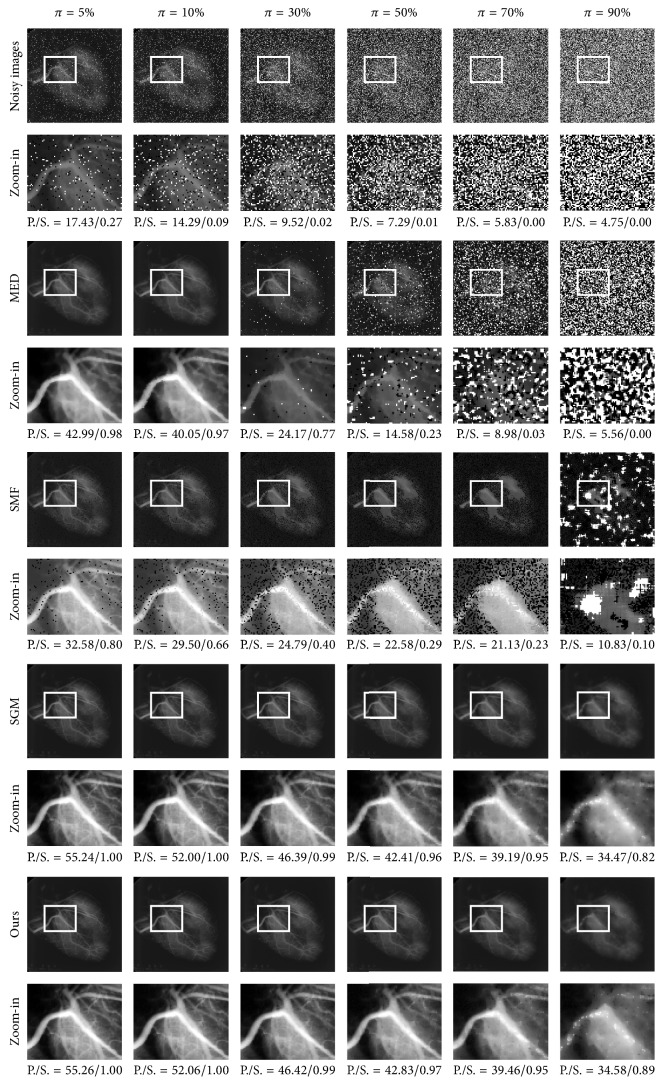
Visual results from different methods on the Heart image in SPM.

To augment the performance evaluations, the proposed SPM method was compared with MED [1], WMF [3], SMF [6], RAMF [31], and SGM [7]. The MED filter used the sliding window of size 3 × 3. The WMF duplicated the current pixel twice and each of the 4 neighbors duplicated itself once, while the pixels on the diagonal were not duplicated, in a window of size 3 × 3. The SMF used the median-based impulse detector, with a window of size 13 × 13 and selecting determining threshold value 70. In the Rank-Ordered Adaptive Median Filter (RAMF), when noise densities were 0.05, 0.1, 0.2, 0.3, 0.4, 0.5, 0.6, 0.7, 0.8, and 0.9, the maximum window radius values were set to 1, 2, 3, 4, 4, 5, 5, 6, and 6, respectively. The source codes of SGM were taken from the original authors [7], and the parameters used in the experiments are those recommended by the authors. The five methods were applied on the same test set Γ. Along with the proposed method in SPM and the noisy method, the PSNR/SSIM results from the seven methods are presented in Table 8, and the highest PSNR/SSIM result for each method and on each noise level is highlighted in bold. The noisy method means that the metrics in (37) and (38) employ noisy images *u*_0_, rather than restored images $\hat{u}$. Apart from the comparative PSNR/SSIM results, visual comparison results for the Barbara and Heart images are shown in Figures 10 and 11, respectively, and the information about these images is labeled sideways.

From the quantitative measurement and visual results in SPM, we make the following observations and conclusions. Firstly, the proposed method achieved the highest PSNR and also the highest SSIM in almost every case. The proposed method achieved a 0.12 dB improvement over the SGM method on average and significantly outperformed the RAMF, the SMF, the WMF, and the MED filter by 6.35 dB, 14.11 dB, 17.89 dB, and 16.49 dB on average, respectively, in the PSNR results. The proposed method achieved a 0.008 improvement over the SGM method on average and significantly outperformed the RAMF, the SMF, the WMF, and the MED filter by 0.097, 0.379, 0.443, and 0.428 on average, respectively, in the SSIM results. Secondly, the proposed method has strong capability to preserve details. It reconstructed more image details from the noisy image than the RAMF, the SMF, the WMF, and the MED filter and was also superior to the SGM method. In particular, the tumor in the breast is clearly visible in the restored image produced by the proposed method, even though the Breast image was corrupted by very high noise, as can be seen in Figure 9. Thirdly, the proposed method was more robust to different noise strengths than the five comparative methods.

**Figure 12:**
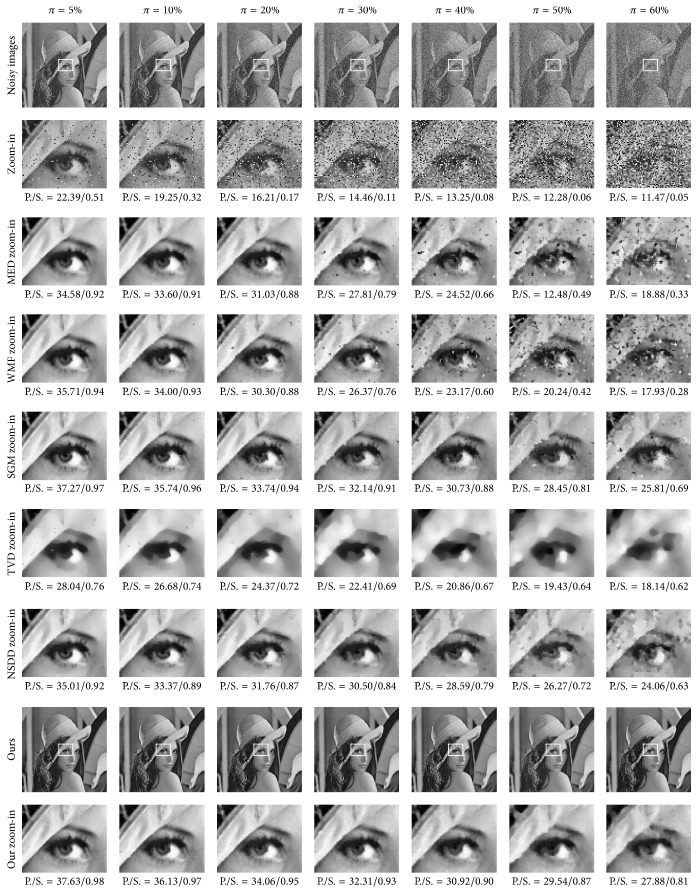
Visual results from different methods on the Lena image in RDM.

*Experimental Results and Comparisons in RDM*. By employing the twenty-one original noise-free images, another test set was built for the proposed method in RDM, denoted by Ψ = {Ψ_1_, Ψ_2_, Ψ_3_}. In each group Ψ_*k*_, the noisy versions of seven images were with different densities of random valued impulse noise. Using the parameter values shown in the RDM field in Table 4, the proposed RDM method was applied to the test set Ψ; the corresponding PSNR and SSIM results are reported in Table 9. The visual results associated with the Lena image are shown in Figure 12.

For comparisons, another two close methods were applied on the test set Ψ. One is the TVD method (see (6)), where the noise-free and noisy pixels are processed uniformly. The other is the NSDD (see (10)) [23]. TVD uses a 0.5 time step size. The NSDD parameters were those recommended by the authors. Apart from the close methods, three additional filters, MED [1], WMF [3], and SGM [7], were appended for comparisons. They were also applied on the same test set Ψ. The MED filter used the sliding window of size 3 × 3. The WMF duplicated the current pixel twice and each of the 4 neighbors duplicated itself once, while the pixels on the diagonal were not duplicated, in a window of size 3 × 3. The source codes of SGM were taken from the original authors [7], and the parameters used in the experiments are those recommended by the authors. The PSNR and SSIM results of the five methods are also reported in Table 9, and visual results associated with the Lena image are also shown in Figure 12.

In addition, the mean PSNR and mean SSIM results for fixed noise are calculated for noisy images, the MED filter, WMF, SGM, TVD, NSDD, and the proposed method in RDM, respectively. The corresponding calculation is as follows:

(39) PSNR−π=1Ψπ∑k∈ΨπPSNRπk,

(40) SSIM−π=1Ψπ∑k∈ΨπSSIMπk.

**Figure 13:**
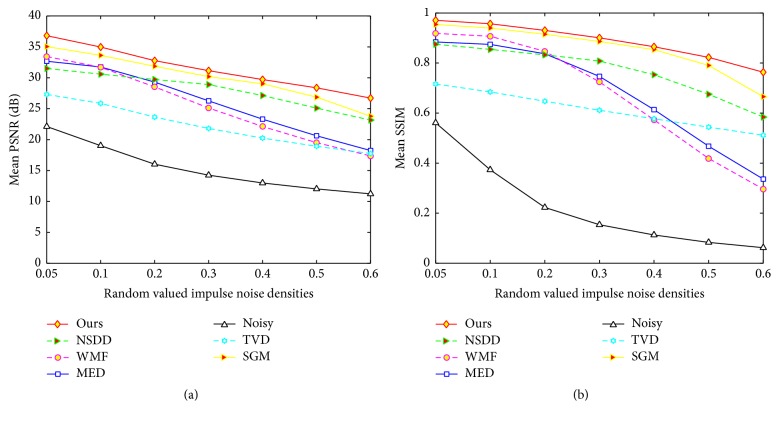
The mean PSNR and SSIM results for fixed noise from different RDM methods.

In the above two formulae, Ψ_*π*_ denotes all the noisy images with the same density noise *π* in Ψ, and PSNR_*π*_(*k*) and SSIM_*π*_(*k*) denote the PSNR and SSIM values of the *k*th image with the noise of density *π*, respectively. For example, if (39) is applied to the proposed RDM method, ${\overset{-}{PSNR}}_{0.05}$ denotes the mean PSNR of the twenty-one images on the 0.05 density noise. The mean values of the two metrics on different noise levels are plotted in Figure 13.

We draw the following observations and conclusions from the quantitative measurements and visual results in RDM. Firstly, the proposed method achieved the best results in every case tested. The proposed method achieved 1.441 dB improvement over the SGM method on average and significantly outperformed the NSDD, MED, WMF, TVD, and the noisy method by 3.486 dB, 5.478 dB, 6.106 dB, 9.283 dB, and 16.123 dB on average, respectively, in the PSNR results. The proposed method achieved 0.029 improvement over the SGM method on average and significantly outperformed the NSDD, MED, WMF, TVD, and the noisy method by 0.118, 0.207, 0.218, 0.274, and 0.663 on average, respectively, in the SSIM results. Secondly, the proposed method has a strong capability to preserve detail. The proposed method reconstructed more image details from noisy images than SGM, NSDD, TVD, WMF, and MED. A few strong noisy pixels appeared in the filtered image by the SGM method. The NSDD method created ring artifacts on smooth regions; the TVD method introduced too much smoothing and resulted in blurred visual features; the MED filter and the WMF worked quite well on low noise but faltered on high noise. Thirdly, the proposed method was more robust to noise density than the five comparative methods.

In summary, the proposed method shows strong capability of reducing noise not only in SPM but also in RDM, in terms of the PSNR/SSIM results. In addition, it produced a higher visual perception quality in restored images, in comparison with results from other methods.

### 4.4. Comparison of Computational Complexity in RDM

The computational complexity of the proposed method was compared with the NSDD method in RDM. Both denoising structures consist of two stages: detection and removal. As an analogy, calculating ENI_*P*_(*u*, *w*, *T*) in (10) is equivalent to obtaining the determining function *h*(*x*, *y*) in (17), and the flow of NSDD in (10) is equivalent to the flow in (22). However, there exist some differences. The function *h*(*x*, *y*) is calculated only once, while ENI_*P*_(*u*, *w*, *T*) need to be updated once per iteration; the flow in (22) is for a part of an image domain, while the flow in (10) is for all pixels in the image domain; the number of iterations in the proposed method is larger than in the NSDD method. From this analysis, the computation time of the proposed method is shown to be shorter than that of NSDD during iteration, but the total computation time was longer because the proposed method needs more iterations than NSDD. The Monarch image was tested, with 0.05 density noise. The proposed method took 0.5837 seconds for an iteration, while the NSDD took 1.8258 seconds; the proposed method took 62.4213 seconds to yield a restored Monarch image, while the NSDD took 13.4974 seconds, using Matlab-R2012b implementation on eight machines with Intel(R) Core(TM) i7-4702MQ CPU at 2.20 GHz.

## 5. Conclusions

In this paper, a new method is proposed to reduce impulsive noise for grayscale images. The proposed method consists of two schemes associated with the salt-and-pepper noise and random valued impulse models, respectively. In the salt-and-pepper noise model, the detector uses two thresholds (*T*, thr) to divide pixels into corrupted and noise-free. If a pixel is declared as corrupted, modified total variation diffusion is applied; otherwise, the pixel is left unchanged. In addition, an acceleration method is proposed to reduce the computation cost for the salt-and-pepper noise model. The initial image for the iterative process is an evolved intermediate image, rather than a noisy image. In the random valued impulse model, a ROAD detector uses three thresholds (*α*, *T*_1_, *T*_2_) to divide pixels into corrupted, noise-free, and possibly corrupted. If a pixel is declared as corrupted, modified total variation diffusion is applied; if it is declared as a noise-free pixel, weighted total variation diffusion is applied; otherwise, the pixel is left unchanged. In experiments, two test sets (Γ, Ψ) associated with the two noise models were built for evaluations of the proposed method. Experimental results show that the proposed method is robust for different strength noise; for example, it can restore the images corrupted by salt-and-pepper noise whose densities vary from 5% to 90% and can restore the images corrupted by random valued impulse whose densities vary from 5% to 60%. Moreover, the proposed method exhibits strong capabilities for noise removal in terms of PSNR/SSIM results and the visual perception quality of restored images.

## Figures and Tables

**Table 1:** The desirable number of iterations before and after acceleration when Δ*t* = 0.8.

*π*	10%	20%	30%	40%	50%	60%	70%	80%	90%
Before	103	120	142	158	180	250	278	370	700
After	27	30	40	41	43	66	69	125	145

**Table 2:** The value of the parameter *R*^0^ in SPM.

*π*	10%	20%	30%	40%	50%	60%	70%	80%	90%
*R* ^0^	1	2	3	4	4	5	5	6	6

**Table 3:** The value of the parameter *N* used in RDM.

*π*	5%	10%	20%	30%	40%	50%	60%
*N*	120	135	170	210	230	300	550

**Table 4:** The parameters used in the proposed method.

Model	Module	Parameters and comments
SPM	Detection	*R* = 2. The neighborhood radius.
		*T* = 60. The threshold for judging the relationship between a pixel and its neighbors.
		thr = 3. The threshold that decides whether or not a pixel is corrupted.
	Removal	Δ*t* = 0.8. The time marching step size.
		*N*: Table 1. The desirable number of iterations.
	Acceleration	*R* ^0^: Table 2. The filtering window radius.

RDM	Detection	*R* = 2. The neighborhood radius.
		*α* = 14. The threshold used in *α*-trimmed ROAD statistic.
		*T*_1_ = 150. The threshold that decides whether a pixel is not corrupted.
		*T*_2_ = 320. The threshold that decides whether a pixel is corrupted.
	Removal	Δ*t* = 0.5. The time marching step size.
		*N*: Table 3. The desirable number of iterations.

**Table 5:** The PSNR/SSIM results for miscellaneous images in SPM.

*π*	Test images						
	Monarch	Lena	House	Couple	Cameraman	Boat	Barbara
10%	39.56/0.99	44.28/0.99	44.66/0.99	40.23/0.99	36.59/0.99	39.91/0.98	35.09/0.98
20%	34.78/0.99	40.42/0.98	40.13/0.98	36.43/0.97	32.39/0.97	36.22/0.97	31.49/0.97
30%	31.94/0.98	37.89/0.97	37.56/0.97	33.97/0.96	30.09/0.96	33.86/0.95	29.51/0.94
40%	29.50/0.96	35.78/0.96	35.14/0.95	31.98/0.93	28.23/0.93	31.96/0.92	27.95/0.91
50%	27.74/0.94	34.07/0.94	33.33/0.93	30.29/0.90	26.68/0.90	30.35/0.90	26.69/0.88
60%	25.89/0.91	32.49/0.92	31.72/0.91	28.79/0.87	25.50/0.87	28.88/0.86	25.57/0.84
70%	24.16/0.87	30.68/0.89	29.76/0.87	27.22/0.81	24.07/0.83	27.27/0.81	24.62/0.79
80%	22.34/0.81	28.87/0.84	27.86/0.83	25.56/0.74	22.70/0.77	25.57/0.74	23.67/0.73
90%	19.52/0.67	26.20/0.77	24.86/0.75	23.43/0.61	20.84/0.68	23.43/0.63	22.53/0.64

**Table 6:** The PSNR/SSIM results for texture images in SPM.

*π*	Test images						
	Roof	Rooftiles	Fence	Brickwall	Fingerprint	Straw	Brick
10%	40.12/0.98	40.97/0.99	42.43/0.98	40.62/0.98	40.53/1.00	40.89/0.99	39.75/0.98
20%	36.62/0.96	37.42/0.98	39.33/0.97	37.35/0.96	36.49/0.99	37.63/0.98	36.34/0.96
30%	34.16/0.94	34.98/0.96	37.43/0.95	35.18/0.94	33.66/0.98	35.51/0.97	34.24/0.93
40%	32.12/0.91	32.96/0.95	36.02/0.93	33.40/0.92	31.19/0.97	33.73/0.95	32.54/0.90
50%	30.38/0.88	31.17/0.92	34.81/0.90	31.88/0.89	28.91/0.94	32.02/0.93	31.09/0.87
60%	28.62/0.84	29.41/0.90	33.66/0.88	30.35/0.85	26.67/0.91	30.27/0.90	29.70/0.82
70%	26.61/0.78	27.15/0.86	32.23/0.84	28.48/0.79	23.97/0.84	28.05/0.85	28.28/0.75
80%	24.45/0.69	24.38/0.79	30.33/0.78	26.14/0.71	20.93/0.71	25.29/0.76	26.77/0.67
90%	22.37/0.56	20.14/0.61	27.25/0.64	23.01/0.55	17.61/0.42	21.58/0.55	24.78/0.54

**Table 7:** The PSNR/SSIM results for biomedical images in SPM.

*π*	Test images						
	Algae	Breast	C05c	Cellulas	Crmo4280	Heart	Muscle
10%	44.80/0.98	30.38/0.99	46.35/0.99	43.09/0.99	51.31/1.00	52.06/1.00	39.87/0.99
20%	41.47/0.96	29.48/0.99	42.92/0.98	39.51/0.97	47.44/0.99	48.73/0.99	35.95/0.98
30%	39.63/0.94	29.47/0.99	41.03/0.97	37.52/0.95	44.80/0.99	46.42/0.99	33.18/0.97
40%	38.25/0.91	29.46/0.98	39.43/0.95	35.95/0.93	42.76/0.98	44.64/0.98	31.15/0.95
50%	36.96/0.89	29.38/0.97	38.17/0.93	34.64/0.91	40.96/0.97	42.83/0.97	29.56/0.93
60%	35.90/0.86	29.32/0.96	37.01/0.92	33.37/0.88	39.15/0.95	41.36/0.96	27.70/0.91
70%	34.96/0.84	29.17/0.94	35.81/0.89	32.06/0.84	37.42/0.93	39.46/0.95	26.00/0.87
80%	33.36/0.80	28.99/0.91	34.33/0.86	30.48/0.79	35.37/0.89	37.38/0.92	24.15/0.83
90%	30.96/0.77	28.58/0.87	32.47/0.81	28.06/0.70	32.83/0.83	34.58/0.89	21.25/0.74

**Table 8:** The PSNR/SSIM results from different methods in SPM.

Methods	Salt-and-pepper noise density *π*					
	5%	10%	30%	50%	70%	90%
Miscellaneous image: Barbara						
Noisy	18.31/0.44	15.25/0.27	10.49/0.10	8.26/0.05	6.81/0.02	5.72/0.01
MED	25.10/0.81	24.66/0.79	20.89/0.63	14.54/0.24	9.66/0.06	6.42/0.01
WMF	27.06/0.88	26.07/0.86	19.78/0.60	13.44/0.20	9.19/0.05	6.31/0.01
SMF	28.56/0.90	27.02/0.85	23.57/0.70	21.56/0.60	19.49/0.48	11.00/0.13
RAMF	29.25/0.94	28.74/0.93	26.29/0.89	24.01/0.81	21.88/0.69	13.80/0.29
SGM	38.53/**0.99**	34.99/**0.98**	29.27/**0.94**	26.49/**0.88**	24.54/**0.79**	22.44/**0.64**
Ours	**38.74/0.99**	**35.09/0.98**	**29.51/0.94**	**26.69/0.88**	**24.62/0.79**	**22.53/0.64**

Texture image: Straw						
Noisy	18.15/0.54	15.08/0.33	10.30/0.11	8.07/0.05	6.61/0.02	5.52/0.01
MED	31.73/0.92	30.55/0.90	22.60/0.76	14.74/0.37	9.58/0.11	6.24/0.02
WMF	32.67/0.94	31.02/0.93	20.72/0.71	13.56/0.31	9.08/0.09	6.14/0.02
SMF	27.86/0.90	25.82/0.84	21.75/0.64	19.55/0.50	17.66/0.36	10.62/0.10
RAMF	36.27/0.97	35.28/0.96	30.90/0.93	27.28/0.85	23.61/0.72	13.73/0.32
SGM	44.01/**1.00**	40.72/**0.99**	35.45/**0.97**	32.02/0.92	28.04/0.81	21.50/0.52
Ours	**44.03/1.00**	**40.89/0.99**	**35.51/0.97**	**32.02/0.93**	**28.05/0.85**	**21.58/0.55**

Biomedical image: Heart						
Noisy	17.43/0.27	14.29/0.09	9.52/0.02	7.29/0.01	5.83/0.00	4.75/0.00
MED	42.99/0.98	40.05/0.97	24.17/0.77	14.58/0.23	8.98/0.03	5.56/0.00
WMF	34.95/0.98	31.53/0.98	19.86/0.62	12.81/0.14	8.35/0.02	5.39/0.00
SMF	32.58/0.80	29.50/0.66	24.79/0.40	22.58/0.29	21.13/0.23	10.83/0.10
RAMF	47.39/0.99	46.79/0.99	42.94/0.98	39.17/0.96	33.93/0.92	14.37/0.43
SGM	55.24/**1.00**	52.00/**1.00**	46.39/**0.99**	42.41/0.96	39.19/**0.95**	34.47/0.82
Ours	**55.26/1.00**	**52.06/1.00**	**46.42/0.99**	**42.83/0.97**	**39.46/0.95**	**34.58/0.89**

**Table 9:** The PSNR/SSIM results from different methods in RDM.

Methods	Random valued impulse noise density *π*						
	5%	10%	20%	30%	40%	50%	60%
Miscellaneous image: Monarch							
Noisy	21.96/0.62	18.88/0.46	15.92/0.31	14.14/0.23	12.82/0.17	11.86/0.13	11.08/0.10
MED	29.97/0.95	28.80/0.94	26.46/0.90	24.17/0.82	21.79/0.69	19.55/0.54	17.42/0.40
WMF	31.95/0.97	29.77/0.96	26.59/0.90	23.63/0.79	20.87/0.65	18.55/0.49	16.62/0.36
SGM	30.80/0.97	29.30/0.96	27.43/0.93	26.14/0.91	25.30/0.88	23.43/0.81	21.09/0.71
TVD	25.76/0.79	24.04/0.77	21.89/0.71	20.19/0.67	18.61/0.61	17.38/0.55	16.33/0.50
NSDD	29.54/0.92	27.65/0.88	26.73/0.87	25.42/0.85	23.83/0.79	22.00/0.72	20.71/0.64
Ours	31.00/0.97	29.49/0.96	27.70/0.94	26.16/0.91	24.83/0.88	23.26/0.84	21.52/0.78

Miscellaneous image: Lena							
Noisy	22.39/0.51	19.25/0.32	16.21/0.17	14.46/0.11	13.25/0.08	12.28/0.06	11.47/0.05
MED	34.58/0.92	33.60/0.91	31.03/0.88	27.81/0.79	24.52/0.66	21.48/0.49	18.88/0.33
WMF	35.71/0.94	34.00/0.93	30.30/0.88	26.37/0.76	23.17/0.60	20.24/0.42	17.93/0.28
SGM	37.27/0.97	35.74/0.96	33.74/0.94	32.14/0.91	30.73/0.88	28.45/0.81	25.18/0.69
TVD	28.04/0.76	26.68/0.74	24.37/0.72	22.41/0.69	20.86/0.67	19.43/0.64	18.14/0.62
NSDD	35.01/0.92	33.37/0.89	31.76/0.87	30.50/0.84	28.59/0.79	26.27/0.72	24.06/0.63
Ours	37.63/0.98	36.13/0.97	34.06/0.95	32.31/0.93	30.92/0.90	29.54/0.87	27.88/0.81

Texture image: Fence							
Noisy	23.48/0.59	20.39/0.38	17.38/0.21	15.60/0.13	14.35/0.09	13.37/0.06	12.57/0.05
MED	33.26/0.84	32.76/0.84	31.39/0.81	29.54/0.76	27.09/0.67	24.48/0.55	22.06/0.41
WMF	34.15/0.88	33.26/0.87	31.19/0.84	28.36/0.76	25.60/0.64	22.97/0.49	20.76/0.35
SGM	37.46/0.95	36.11/0.93	34.17/0.91	32.73/0.87	31.33/0.84	29.45/0.79	26.47/0.67
TVD	28.30/0.65	27.54/0.62	26.45/0.58	25.42/0.55	24.54/0.52	23.73/0.49	22.95/0.46
NSDD	34.33/0.89	33.34/0.86	32.00/0.82	31.03/0.79	29.45/0.74	27.76/0.68	26.09/0.60
Ours	41.05/0.98	38.77/0.97	36.24/0.94	34.50/0.91	33.08/0.87	31.68/0.83	30.25/0.77

Texture image: Straw							
Noisy	21.41/0.68	18.39/0.50	15.37/0.31	13.61/0.22	12.36/0.16	11.38/0.11	10.59/0.08
MED	31.75/0.92	30.70/0.90	28.24/0.87	25.19/0.79	21.96/0.67	19.12/0.53	16.71/0.40
WMF	32.95/0.94	31.54/0.93	28.09/0.87	24.30/0.78	21.01/0.64	18.22/0.49	16.04/0.36
SGM	34.92/0.97	33.34/0.95	31.32/0.93	29.69/0.91	28.29/0.88	26.15/0.82	22.67/0.71
TVD	24.64/0.69	23.29/0.62	21.31/0.56	19.60/0.51	18.10/0.46	16.76/0.41	15.60/0.37
NSDD	33.10/0.94	31.18/0.92	29.16/0.88	27.50/0.84	25.15/0.77	22.79/0.68	20.91/0.59
Ours	37.68/0.98	35.22/0.97	32.71/0.95	30.97/0.92	29.36/0.89	27.53/0.85	25.17/0.77

Biomedical image: Algae							
Noisy	23.62/0.46	20.48/0.25	17.48/0.12	15.68/0.07	14.42/0.05	13.47/0.04	12.68/0.03
MED	35.20/0.81	34.85/0.81	33.33/0.79	30.63/0.73	27.75/0.63	25.16/0.49	22.51/0.33
WMF	35.75/0.85	34.13/0.84	31.96/0.80	28.76/0.71	25.87/0.57	23.33/0.40	20.94/0.26
SGM	39.92/0.95	38.59/0.93	36.42/0.90	34.91/0.86	33.68/0.83	31.16/0.76	27.59/0.63
TVD	32.94/0.76	31.84/0.76	30.26/0.75	28.51/0.74	27.04/0.74	25.98/0.73	24.83/0.72
NSDD	35.44/0.83	35.56/0.83	34.02/0.79	34.06/0.78	32.61/0.76	30.71/0.71	28.84/0.64
Ours	42.75/0.98	40.85/0.96	38.55/0.93	37.04/0.91	35.52/0.87	34.17/0.83	32.60/0.79

Biomedical image: Heart							
Noisy	19.97/0.33	16.99/0.15	13.90/0.06	12.16/0.03	10.88/0.02	9.91/0.02	9.14/0.01
MED	43.10/0.98	40.84/0.97	33.80/0.89	26.19/0.66	20.96/0.38	17.41/0.20	14.61/0.11
WMF	36.79/0.98	33.59/0.97	27.52/0.83	22.59/0.55	18.93/0.31	15.85/0.16	13.74/0.09
SGM	47.29/0.99	44.91/0.99	42.44/0.97	37.66/0.95	36.33/0.92	30.06/0.85	24.40/0.68
TVD	30.67/0.84	27.47/0.82	22.60/0.76	19.40/0.70	17.11/0.64	15.14/0.60	13.60/0.56
NSDD	30.91/0.85	32.54/0.85	34.98/0.87	34.59/0.86	30.93/0.79	26.90/0.66	23.06/0.50
Ours	48.97/0.99	46.79/0.99	43.71/0.98	41.65/0.97	39.37/0.95	38.04/0.93	35.07/0.91
